# Palaeoenvironmental Shifts Drove the Adaptive Radiation of a Noctuid Stemborer Tribe (Lepidoptera, Noctuidae, Apameini) in the Miocene

**DOI:** 10.1371/journal.pone.0041377

**Published:** 2012-07-31

**Authors:** Emmanuel F. A. Toussaint, Fabien L. Condamine, Gael J. Kergoat, Claire Capdevielle-Dulac, Jérôme Barbut, Jean-François Silvain, Bruno P. Le Ru

**Affiliations:** 1 Department of Entomology, Zoological State Collection, Munich, Germany; 2 Centre National de la Recherche Scientifique, Centre de Mathématiques Appliquées (Ecole Polytechnique), Palaiseau, France; 3 Institut National de la Recherche Agronomique, Unité Mixte de Recherche Centre de Biologie pour la Gestion des Populations (Institut National de la Recherche Agronomique, Institut de Recherche pour le Développement, Centre de coopération internationale en recherche agronomique pour le développement, Montpellier SupAgro), Campus International de Baillarguet, Montferrier-sur-Lez, France; 4 Institut de Recherche pour le Développement, Laboratoire Evolution, Génomes et Spéciation, Centre National de la Recherche Scientifique, Gif-sur-Yvette, France and Université Paris, Orsay, France; 5 Département Systématique et Évolution, Entomologie, Muséum national d’Histoire naturelle, Paris, France; 6 Institut de Recherche pour le Développement, International Centre of Insect Physiology and Ecology, Nairobi, Kenya and Université Paris, Orsay, France; CNRS/Université Joseph-Fourier, France

## Abstract

Between the late Oligocene and the early Miocene, climatic changes have shattered the faunal and floral communities and drove the apparition of new ecological niches. Grassland biomes began to supplant forestlands, thus favouring a large-scale ecosystem turnover. The independent adaptive radiations of several mammal lineages through the evolution of key innovations are classic examples of these changes. However, little is known concerning the evolutionary history of other herbivorous groups in relation with this modified environment. It is especially the case in phytophagous insect communities, which have been rarely studied in this context despite their ecological importance. Here, we investigate the phylogenetic and evolutionary patterns of grass-specialist moths from the species-rich tribe Apameini (Lepidoptera, Noctuidae). The molecular dating analyses carried out over the corresponding phylogenetic framework reveal an origin around 29 million years ago for the Apameini. Ancestral state reconstructions indicate (i) a potential Palaearctic origin of the tribe Apameini associated with a major dispersal event in Afrotropics for the subtribe Sesamiina; (ii) a recent colonization from Palaearctic of the New World and Oriental regions by several independent lineages; and (iii) an ancestral association of the tribe Apameini over grasses (Poaceae). Diversification analyses indicate that diversification rates have not remained constant during the evolution of the group, as underlined by a significant shift in diversification rates during the early Miocene. Interestingly, this age estimate is congruent with the development of grasslands at this time. Rather than clade ages, variations in diversification rates among genera better explain the current differences in species diversity. Our results underpin a potential adaptive radiation of these phytophagous moths with the family Poaceae in relation with the major environmental shifts that have occurred in the Miocene.

## Introduction

One of the most striking features of biodiversity on Earth lies in the fact that some lineages are way more species-rich than others [1]. Previous works supported that the variation in species richness between groups may be explained either by clade ages [2], [3] or differences in per-lineage rates of speciation and extinction [4]. Pioneer studies have also tested for variation in diversification rates on large taxonomic scales (see [5] on angiosperms, [6] on birds, [2] on Coleoptera), but it is only recently that some works have addressed the extent to which diversification rates vary within species-level radiations [7], [8]. Understanding the contribution of time and/or diversification rate variations to differences in species richness requires more empirical studies. To address these questions, the order Lepidoptera that ranks second in term of species richness (behind Coleoptera) among insects is particularly suitable.

Moths from the tribe Apameini (Lepidoptera, Noctuidae) are distributed worldwide except in the Neotropics, especially in grass-dominated wet ecotones. This group comprises more than 800 species that are traditionally gathered into 93 recognized genera and three subtribes; Apameina, Oxytripiina and Sesamiina [9], [10]. Interestingly, most species from the Apameina (about 600 species) are found in the Holarctic region, whereas most species of Sesamiina (about 200 species) are distributed in the Afrotropical region [11], [12]. Besides, the subtribe Oxytripiina comprises only two western Palaearctic species *Oxytripia orbiculosa* and *O. stephania*. Within Apameini, most of the caterpillars are true endophagous larvae and present a stem-boring, rhizome-boring or cut-worming behaviour [9]. Key adaptations such as the enlargement of mandibular muscles for foraging silica-rich stalks [9], but also the endophagy that provides a protection against external biotic and abiotic conditions [13], emphasize the close association of those moths with their host-plants. Although their evolutionary history is poorly understood, the diversification of Apameini seems to be recent and linked to the major climatic changes experienced during the late Neogene [14], [15]. However, this hypothesis has only been put forward for the Sesamiina clade and remains to be tested for the whole tribe [14], [15]. Moreover, it appears that the radiation of the tribe is closely tied to the evolutionary history of grasses on which the majority of species are specialized [9], [16], [17]. Hence, the tribe Apameini represents a relevant model to (*i*) test the extent to which diversification rates vary among lineages during continental evolutionary radiations, and (*ii*) test the association between a phytophagous insect tribe and grasses (mainly Poaceae), as a result of wide climate changes during the Cenozoic, such as the Oligocene-Miocene transition.

Specifically, the Oligocene-Miocene boundary (around 23.0 million years [Myr] ago) is generally known for having contributed to shape a major turnover of the ecosystems in the low and middle latitudes of the planet mostly due to the marked fluctuations in atmospheric CO_2_ values [18]. Those variations combined with a global climate cooling [19] and an expanding aridity have led to progressive but deep changes over the terrestrial biomes [20], [21]. Woodlands and tropical forests from the Early Cenozoic receded in northern areas and gave way to more open landscapes such as grass-dominated habitats like steppes and savannahs which are better adapted to seasonal extremes of moisture and temperate fluctuations [20], [22]. The main changes of floral communities associated with a lower primary productivity and the transition from C_3_ to C_4_ plants during the early Neogene are thought to have deeply influenced the evolution of faunal communities [21], [23]. It is particularly true for phytophagous lineages that progressively adapted and diversified in these new ecological niches [8], [24].

Thanks to the abundant fossil records, the role of grasslands in the evolution of mammals is well documented; herbivorous leaf eaters declined in favour of grass eaters having developed key innovations in terms of teething and plant assimilation at the same time in different lineages [20], [25], [26]. This turnover pattern, combined with adaptive evolutions, is also observed in forest-adapted groups like marsupials where the ubiquity of grasslands led to the decline of many lineages and the apparition of more adapted taxa such as grazing kangaroos [27]. Although poorly documented, many phytophagous insects adapted to grasslands through the evolution of key innovations [28], [29], [30]. The hemipteran family Cicadellidae (25,000 species) exhibits more than 8,000 species associated with grasslands, which appear to have diversified during the expansion of major grasslands biomes [31]. Butterflies of the tribe Satyrini (2,200 species) seem to have originated before grasses, but mostly diversified during the expansion of grasslands biomes, indicating a potential adaptive radiation with grasslands [32]. Likewise, the late Neogene spreading of their host-plants (Poaceae grasses) might have influenced the diversification of pollen feeding beetles from the subtribe Anisopliina [33]. The results of these studies pave the way for supplementary researches on phytophagous insects in the field of co-evolutionary events. However, all these studies have not relied on the use of diversification rates to test whether high species diversity within a group is the outcome of increased diversification rates within a genus, or whether it reflects a decline in diversification elsewhere in the radiation.

Here, we aim to (*i*) infer the first genus-level phylogenetic relationships for the Apameini with reference to the placement of the two main subtribes using molecular data; (*ii*) estimate divergence times using a relaxed molecular-clock approach with fossil and geological calibrations to test hypothesis of ancient versus recent diversification and investigate the level of correlation between the diversification of the Apameini and the apparition of grassland biomes; (*iii*) reconstruct the shifts in host-plant preferences and biogeographic regions using maximum likelihood optimizations; and (iv) test, using a likelihood framework based on the birth-death process [34], whether there is among-lineage variation in diversification rates to explain the high disparities in extant diversity among major groups of this radiation. Finally, we compare our results with data on other groups of phytophagous insects associated with grasslands, in order to discuss the relation between deep changes in biotic communities and the diversification of phytophagous insects during the Oligocene-Miocene transition.

## Materials and Methods

### Taxon Sampling

For this study, since the taxonomy of the tribe Apameini is still debated, the taxon sampling was constructed according to a wide bibliographic compilation in order to only include genera that are clearly described at this taxonomic level and accepted as taxa of this group [9], [11], [12], [14], [35]–[38]. Since Zilli and colleagues are presently working on the taxonomy of the group, we chose in a conservative way not to consider the revision of the *Apamea* species group [10] but instead the latest full revision of the tribe along with other publications in order to avoid any bias in the species-richness inference. Consequently, the sampling comprises a single species for all the genera (13) of the subtribe Sesamiina and more of one-third of the accepted Apameina genera (31 out of 81) [9]. No samples were available for representatives of the subtribe Oxytripiina. A special effort has been made to include the type species of each genus in order to maximize the sampling coverage. Co-authors (BP Le Ru and J Barbut) collected almost all Afrotropical and Palaearctic specimens. No specific permits were required for the described field studies since all the collected species are common and not protected, and the collects took place in unprotected areas. For most of the Nearctic and Oriental specimens, DNA sequences were recovered in the GenBank database (see Table S1 in the electronic supplementary materials for the accession numbers of the tribe).

**Table 1:** Likelihood scores, harmonic means and Bayes factors approximated under Tracer for each partitioning strategy of the MrBayes 3.1.2 analyses for the gene trees.

Gene trees							
	ESS	Likelihood	MrBayes Harmonic mean	SE (1000 rep.)	NoPart	Codon	12v3
Co1NoPart	2027	−12369.890	−12303.52	+/−0.455	–	0	0
Co1Codon	582	−12115.240	−12138.03	+/−0.456	>10	–	0
Co1 12v3	672	−11853.762	−11879.68	+/−0.418	>10	>10	–
EF1NoPart	6136	−9813.363	−9832.57	+/−0.311	–	0	0
EF1Codon	5015	−9513.813	−9534.67	+/−0.373	>10	–	0
EF1 12v3	4353	−9315.471	−9343.53	+/−0.398	>10	>10	–

The Bayes factors are given for each possible comparison of strategies in the three last columns of the table. The analyses with the partitioning strategies ‘12v3’ are the best analyses for both genes (COI and EF1-α). The analysis with the partitioning strategy ‘BySix’ is the best analysis for the combined dataset, and is chosen for the dating analyses as a tree prior.

**Table 2:** Likelihood scores, harmonic means and Bayes factors approximated under Tracer for each partitioning strategy of the MrBayes 3.1.2 analyses for the combined dataset.

Combined dataset								
	ESS	Likelihood	MrBayes Harmonic mean	SE (1000 rep.)	NoPart	ByGene	ByCodon	BySix
NoPart	485	−23880.84	−23904.91	+/−0.464	–	0	0	0
ByGene	1817	−23437.07	−23459.12	+/−0.346	>10	–	>10	0
ByCodon	1509	−23639.10	−23661.91	+/−0.376	>10	0	–	0
BySix	1108	−22871.38	−22891.83	+/−0.425	>10	>10	>10	–

The Bayes factors are given for each possible comparison of strategies in the four last columns of the table. The analysis with the partitioning strategy ‘BySix’ is the best analysis for the combined dataset, and is chosen for the dating analyses as a tree prior.

### Molecular Dataset

For the collected specimens, total DNA was extracted from legs, thoracic or proboscis tissues using Qiagen® DNeasy Animal Tissues kit. After extraction, DNA sequences were obtained using PCR protocols, purification, cleaning and sequencing for the cytochrome oxidase subunit 1 (COI, 666 nucleotides) and elongation-factor alpha (EF-1α, 1239 nucleotides) genes. Sequences were corrected by eye and assembled into consensus under Geneious 5.4 [39], then aligned using Muscle [40]. The coding frame of genes was further checked under Mesquite 2.75 (www.mesquiteproject.org). Special effort has been conducted to add numerous genera from the largest families of Noctuoidea and subfamilies of Noctuidae and Erebidae based on the most recent comprehensive phylogenetic studies of these groups [41], [42]. It is especially true for the family Erebidae for which we sampled diverse genera, more or less derived [42], to allow a more informative use of a fossil at the crown of the clade instead of the stem. Thus, we added 48 genera to the taxon sampling using the GenBank database (see Table S1 in the electronic supplementary materials for the corresponding accession numbers). The species *Nemoria darwiniata* (Dyar) (Geometroidea, Geometridae) was chosen to root the tree, since Geometroidea is recovered as the sister group of Noctuoidea [41].

**Figure 1:**
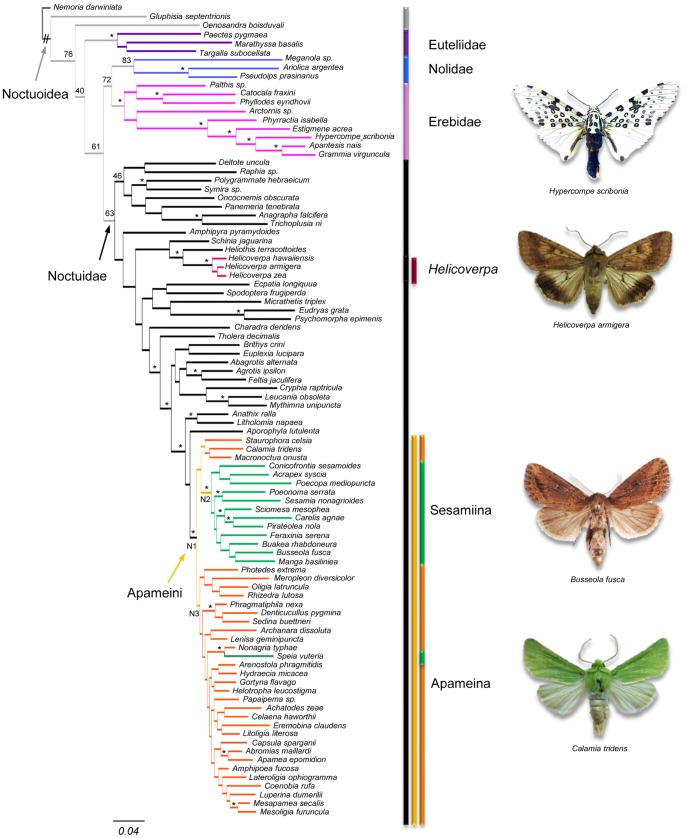
Phylogenetic relationships of the tribe Apameini within the superfamily Noctuoidea under Bayesian inference. Posterior probabilities (PP) are indicated above the nodes (* indicates a value up to 0.95). When no values are indicated, it means that the PP was <0.95, except for some basal nodes for which the support is annotated. Groups of interest are highlighted by vertical bands of different colours referring to the colour of the clades. The Apameini, Sesamiina and Apameina (excluding the three genera *Staurophora*, *Calamia* and *Litholomia*) nodes are respectively labelled N1, N2 and N3. Names of the species for which a habitus is displayed are specified under the pictures (Pictures: EFA Toussaint & BP Le Ru).

### Phylogenetic Inference

Bayesian phylogenetic analyses were performed on each gene using three different strategies of partitioning: (*i*) a unique partition (*NoPart*), (*ii*) a partition by codon position (*ByCodon*), and (*iii*) a partition for the first and second positions against the third one. Such analyses were also realized on the combined dataset using four different strategies of partitioning; (*i*) a unique partition (*NoPart*), (*ii*) a partition for each gene (*ByGene*), (*iii*) a partition by codon position (*ByCodon*), and (*iv*) a partition for each codon position of each gene (*BySix*). For each partition, the substitution models of evolution were defined with jModelTest 0.1.1 [43] using the Bayesian information criterion (BIC; see [44] for a discussion on the rationale of this setting). Bayesian phylogenetic analyses were carried out with MrBayes 3.1.2 [45]. Two independent runs analysed the dataset with eight Markov Chains Monte Carlo (MCMC, one cold and seven incrementally heated) for 2.10^7^ generations, sampling the trees every 1,000^th^ cycle and each MCMC started from a random tree. A conservative burn-in of 25% was applied after checking for stability on the log-likelihood curves and the split-frequencies of the runs (burn-in = 5,000 in this case). All sample trees prior to reaching these generations were discarded and the remaining trees were used to generate a 50% majority rule consensus tree. Additional runs using identical conditions were performed to check that likelihood scores converged on the same posterior distribution of trees. Convergence and selection of the best-fit partitioning strategy in Bayesian analyses were ensured using Bayes Factors (BF; [46]) approximated under Tracer 1.5 (http://tree.bio.ed.ac.uk/software/tracer/).

**Table 3:** Likelihood scores, and Bayes factors approximated under Tracer for the different sets of calibration used in Beast 1.6.2.

	Run statistics			Bayes factors (BF)							
	ESS	Likelihood	SE (1000 rep.)	BD5P Geol	Yule5p Geol	BD5P	Yule5P	BDGeol	Yule Geol	BD	Yule
BD5PGeol	6303	−23880.74	+/−0.24	–	0.481	0.574	0.557	0.432	0.561	0.589	0.429
Yule5PGeol	5785	−23880.01	+/−0.26	2.079	–	1.193	1.158	0.898	1.167	1.123	0.892
BD5P	5814	−23880.19	+/−0.25	1.742	0.838	–	0.971	0.752	0.978	1.025	0.748
Yule5P	4164	−23880.16	+/−0.23	1.794	0.863	1.030	–	0.775	1.007	1.056	0.770
BDGeol	5510	−23879.90	+/−0.22	2.316	1.114	1.329	1.291	–	1.300	1.363	0.994
YuleGeol	4861	−23880.17	+/−0.21	1.782	0.857	1.023	0.993	0.770	–	1.049	0.765
BD	4735	−23880.21	+/−0.21	1.699	0.817	0.975	0.947	0.734	0.954	–	0.729
Yule	6830	−23879.90	+/−0.23	2.330	1.121	1.338	1.299	1.006	1.308	1.371	–

The analysis with the Yule model of speciation with no geological constraint and no corrections for age uncertainties is the best analysis, and is chosen for the main figures in the text.

### Bayesian Estimates of Divergence Times

Divergence-time analyses were performed over the best MrBayes topology with BEAST 1.6.2 [47]. In a preliminary way, the applicability of a molecular clock was investigated for the molecular dataset using PAUP*. Since the hypothesis of a molecular clock was not statistically supported (P<0.05 for all molecular datasets), a method of dating that accounts for rate variation across lineages was used. The .xml file for the BEAST analysis was created under the interface BEAUti (included in the BEAST package) with the following non-default settings and priors: the *Site Model* was set based on the model used in the MrBayes analyses, the *Clock Model* was set to a relaxed-clock with uncorrelated rates, two different *Tree Models* were set separately on independent analyses: a Yule process of speciation and a Birth-Death process of speciation, and the *MCMC parameters* were fixed to 5.10^7^ generations with sampling every 1,000 generations and the first 25% discarded as burn-in. The remaining parameters were left by default. 50% majority rule consensus trees were then generated with the remaining sample trees under TreeAnnotator 1.6.2 (included in the BEAST package). Several independent analyses have been performed to check the likelihood scores under the LogCombiner 1.6.2 (included in the BEAST package) and Tracer programs using the effective sample size criterion [48].

**Figure 2:**
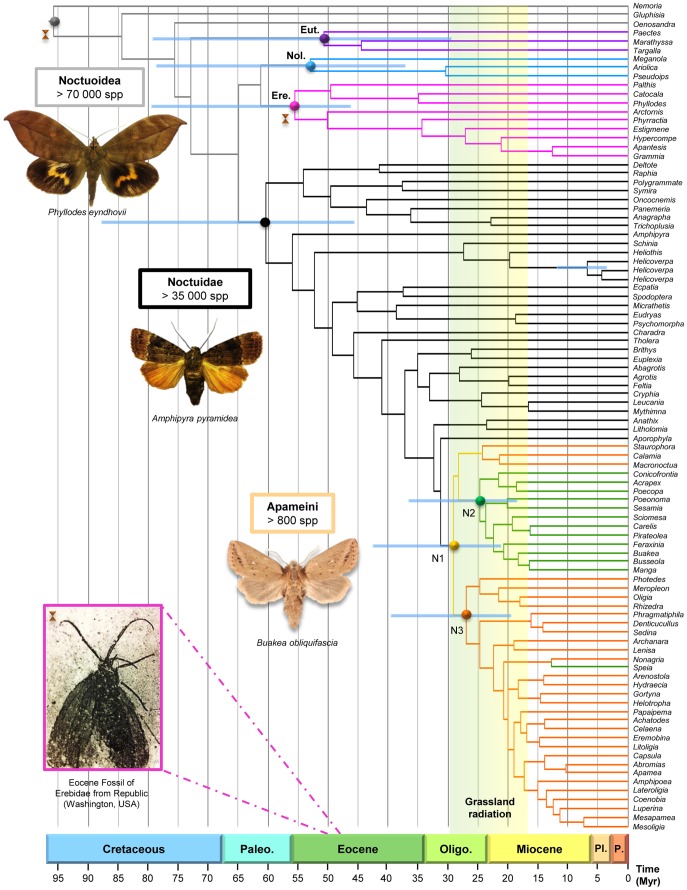
Maximum credibility tree with median ages (Myr) from the Bayesian uncorrelated uniform analysis under BEAST for the superfamily Noctuoidea. A 5 Myr-timescale is placed at the bottom of the chronogram and spans the Cretaceous to the Holocene. Brown hourglasses at the corresponding nodes indicate the calibration points used to ultrametrize the topology. For the fossil calibration, a picture of the moth in question retrieved from Douglas SD & Stockey RA (1996) is presented in the bottom left-corner. Horizontal blue bands represent the 95% HPD heights (in Myr) for the major nodes of the chronogram. Vertical bands and pastilles at the nodes of different colours referring to the colour of the clades highlight groups of interest. For the groups of major interest, the name is given in a box of the clade colour. Abbreviations for the clade names are: Ere.  =  Erebidae, Eut.  =  Euteliidae, Hel.  =  *Helicoverpa*, and Nol.  =  Nolidae. Genera of the Sesamiina and Apameina subtribes show respectively green and orange branches. Names of the species for which a habitus is displayed are specified under the picture (Pictures: EFA Toussaint & BP Le Ru).

No fossil Apameini are known, but thanks to the large taxon sampling assembled for this study, we were able to use three calibration points for the estimation of divergence times. In order to better account for the possible uncertainty of these calibration points, two different methods were used. First, a null model was created in which each calibration point was set with its original value. Second, a smoother model M1 was elaborated in which the lower values of minimum hard bounds were set with the original value decreased by 5% and the upper values of maximum hard bounds were set with the original value increased by 5%. Namely, (*i*) a conservative minimum age of 48.0 (M0)/45.6 (M1) Myr was assigned at the crown of family Erebidae, based on the age of the oldest unambiguous fossil of the group. This fossil (specimen UWBM 66000) is a relatively complete specimen for which a detailed description is available [49]. A discussion of its morphological traits supports its inclusion within the Arctiinae [50]. This fossil was found embedded in tuffs from the Klondike Mountain formation (Republic, Washington State, USA), which were radiometrically dated at approximately 48–49 Myr [51]. All these information were used to set a minimum age at the crown of Erebidae. Then, two maximum ages were used, namely (*i*) a geological constraint and (*ii*) an ecological constraint. The geological constraint relies on the presence of an endemic species in the Hawaiian archipelago, and was assigned at the root of the genus *Helicoverpa* with regard to the presence of *H. hawaiiensis* only known from the entire archipelago. Interestingly, several studies highlighted that before 33 Myr, the archipelago was completely submerged [52], [53], [54]. Between 32 and 8 Myr, a succession of proto-islands exceeding 1000 m emerged in the Hawaiian region before being successively submerged afresh [53]. However, migration flows of biota are thought to have been possible even if very limited, between 8 Myr and the emergence of Kauai 5.1 Myr ago [55], [56], even though the archipelago comprised few emerged islands at this time [53]. In a conservative way we set a maximum age of 33.0 (M0)/34.65 (M1) Myr for the crown of *Helicoverpa*. Finally, we used a maximum age of 183 (M0)/192.15 (M1) Myr corresponding to the origin of Angiosperms (183 Myr; [57]) that was assigned to the root of the tree since it is widely accepted that Lepidoptera could not have emerged before the apparition of flowering plants (see [58], [59] for more rationales on this setting).

**Table 4:** Mean ages and 95% HPD heights for the major nodes of the chronogram (Figure 2).

Median ages in million years (95% HPD heights)								
	With geological calibration				Without geological calibration			
	Uncorrected ages		5% corrected ages		Uncorrected ages		5% corrected ages	
	Yule	Birth-Death	Yule	Birth Death	Yule	Birth-Death	Yule	Birth Death
Root	95.37 (66.42–144.09)	95.85 (66.06–144.35)	91.74 (62.52–142,57)	92.00 (63.19–142.70)	95.74 (67.13–144.05)	95.96 (67.00–145.02)	91.40 (63.00–143.70)	92.52 (63.83–144.61)
Noctuoidea	84.50 (62.57–125.14)	84.39 (62.59–125.20)	81.04 (59.03–123.86)	81.20 (58.90–123.58)	84.34 (62.11–123.54)	84.44 (62.20–125.01)	80.85 (58.02–123.58)	81.20 (58.91–123.66)
Noctuidae	60.54 (46.32–89.56)	60.44 (45.93–89.13)	57.99 (43.39–88.05)	57.99 (42.81–93.69)	60.33 (45.47–88.15)	60.46 (45.46–88.73)	58.09 (43.07–88.64)	58.04 (43.15–88.44)
Erebidae	55.56 (45.97–80.55)	55.47 (46.03–80.59)	53.27 (43.28–79.79)	53.33 (43.31–79.61)	55.49 (45.96–79.94)	55.53 (45.92–80.90)	53.41 (43.36–79.64)	53.22 (43.26–79.53)
Nolidae	53.06 (36.76–79.05)	52.98 (38.85–79.57)	50.79 (35.13–78.84)	50.79 (34.87–78.21)	53.00 (37.39–79.35)	53.08 (37.44–80.11)	50.92 (34.68–78.77)	50.84 (35.23–79.16)
Euteliidae	50.13 (28.18–78.26)	50.18 (28.71–78.64)	48.42 (27.39–77.77)	48.39 (27.50–78.34)	50.63 (29.26–78.78)	51.00 (29.13–80.44)	48.46 (27.96–77.33)	48.81 (28.17–79.13)
Apameini	29.30 21.42–43.43	29.33 (21.34–43.40)	28.22 (20.30–43.07)	28.01 (20.17–42.91)	29.08 (20.96–42.76)	29.19 (21.17–43.34)	28.15 (20.12–43.27)	28.03 (20.07–42.90)
Sesamiina with SMC without *Speia*	28.54 (20.75–42.38)	28.55 (20.93–42.55)	27.45 (19.36–41.70)	27.26 (19.47–41.71)	28.28 (20.64–41.83)	28.41 (20.78–42.34)	27.36 (19.51–42.15)	27.26 (19.71–41.93)
Apameina with *Speia* without SMC	27.15 (19.40–40.37)	27.17 (19.59–40.40)	26.19 (18.62–40.06)	25.94 (18.33–39.83)	26.93 (19.18–39.66)	27.09 (19.66–40.59)	26.10 (18.22–40.09)	25.99 (18.59–40.15)
Sesamiina without SMC without *Speia*	24.97 (17.87–37.25)	24.99 (18.01–37.27)	23.99 (16.98–38.88)	23.83 (19.69–36.43)	24.73 (17.75–36.60)	24.90 (18.06–37.35)	24.01 (16.76–36.99)	23.92 (16.87–36.71)
*Helicoverpa*	6.81 (3.29–11.65)	6.76 (3.38–11.89)	6.47 (3.22–11.52)	6.47 (3.25–11.51)	6.72 (3.31–11.52)	6.80 (3.43–11.76)	6.52 (3.17–11.62)	6.51 (3.23–11.68)

SMC =  Clade comprising the following genera : *Staurophora*, *Macronoctua*, *Calamia*.

Following Ho & Phillips [60] a lognormal distribution was used for the fossil calibration and a uniform distribution for the other constraints. Eventually, accounting for the criticisms regarding the calibrations based on the age of the Hawaiian archipelago and the assumption that it might have represented a “conveyor belt” allowing biodiversity to jump from an older island to a younger one [61], [62], the dating analyses were also carried out without the calibration point based on the emergence of the Hawaiian archipelago.

### Reconstruction of Ancestral Character States (Host-plant Associations and Biogeography)

Ancestral character states of host-plants/areas for Apameini were inferred using the Dispersal-Extinction-Cladogenesis (DEC) model [63] under the program Lagrange (www.code.google.com/p/lagrange/). Although normally used for inferring historical biogeography of groups, the DEC model is relevant for reconstructing other evolution of character states as advocated by Ree & Smith [63]. Therefore, this method was chosen over the classical ML-optimized approaches since it allows (*i*) the use of multiple character states; (*ii*) the optimization of ancestral character states at the root; and (*iii*) the construction of Q matrices permitting the use of transition rates between character states. Specifically for host-plant/area characters, it describes ancestor-descendant transitions between character states by processes of dispersal (host/area colonization), extinction (host/area contraction/specialization), and cladogenesis (host/area inheritance). It specifies the likelihood of species-range data arrayed at the tips of a phylogenetic tree as a function of rates of dispersal and local extinction [63].

**Figure 3:**
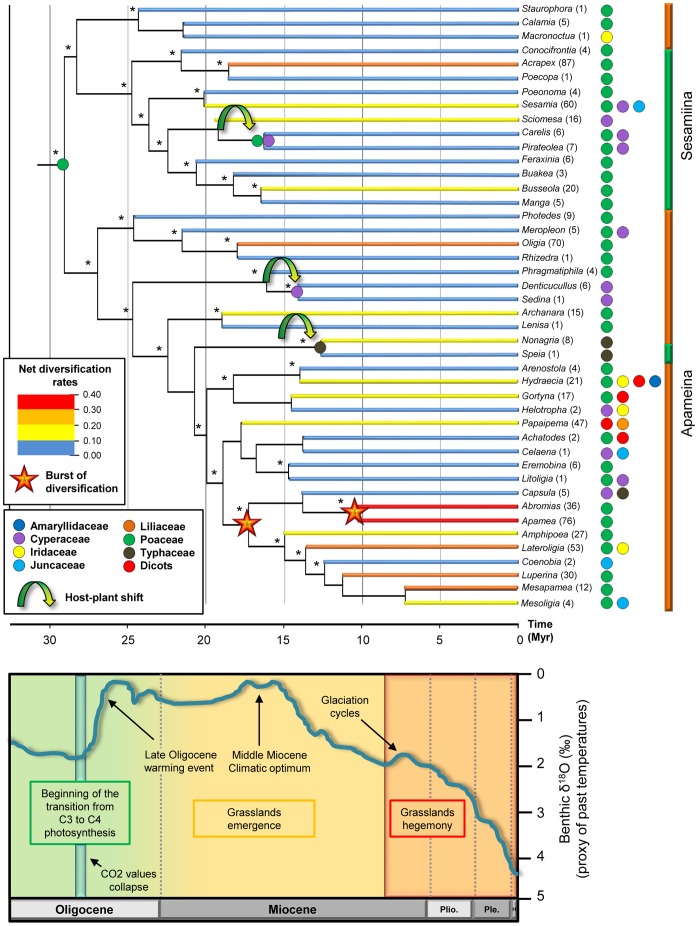
Estimates of net diversification rates and ancestral host plant reconstruction of the tribe Apameini based on the Bayesian chronogram. A coloured pastille indicating the most likely ancestral host plant is displayed at the root of the topology and at the nodes for which a host-plant shift (green arrow) is observed. When no pastille is shown, it means that the ancestral host plant is the same as the root. Present hot-plants are specified on the right of the genera names. Colours of the pastilles correspond to the listed families at the bottom left corner of the chronogram. Asterisks above the nodes indicate a strong support for the recovered family (>2 log-likelihood units against the second better score). A 5 Myr-timescale is placed at the bottom of the chronogram. The vertical bars next to the genera names delimit the two subtribes (Apameina in orange and Sesamiina in green). The net diversification rates calculated for each genus are shown on the branches. Colour of the branches match the different levels of rates presented above the host-plant families. Two stars at corresponding nodes indicate maximum likelihood estimate of rate-shift location inferred under two-rate diversification model. The species richness of each genus is displayed in brackets after the name. At the bottom of the figure, a graphic illustrates the variations in relative temperature (approximated by δ^18^O in ‰) from the mid-Oligocene to the Holocene. Major climatic events are presented along with the most important stages in the evolution of grasses.

An extensive bibliographic survey was realized to compile the larval host-plant preferences [9], [64]. The host-plant associations of species were categorized using the following eight character states: (*i*) Amaryllidaceae; (*ii*) Cyperaceae; (*iii*) Iridaceae; (i*v*) Juncaceae; (*v*) Liliaceae; (*vi*) Poaceae; (*vii*) Typhaceae and (*viii*) Dicotyledons. Species hosts were defined by presence-absence data excluding marginal preference on particular plants. The eight character states yield a set of 2^8^ = 256 theoretically possible host-plant groups (host subsets). In order to decrease computational time, a maximum of three ancestral host-plants was allowed when reconstructing the characters for each node of the topology.

Concerning the distribution of Apameini, the information was retrieved from the literature [9], [11], [16], [17]. The distribution of species was categorized using the following four character states: (*i*) Afrotropical, (*ii*) Nearctic, (*iii*) Oriental and (*iv*) Palaearctic. Similarly to the analysis of host-plant associations, species distributions were defined by presence-absence data excluding marginal distribution on particular areas [65]. Since no geological connexion have ever been found from the Oligocene to be present between Afrotropics and Nearctic and between Oriental and Nearctic, these areas were not considered as adjacent in the analyses [66].

The matrix of host-plant preferences comprised eight host-plant characters, and the matrix of geographic distribution comprised four area characters. Both matrices included the 44 genera of Apameini recovered in the Bayesian chronogram.

**Figure 4:**
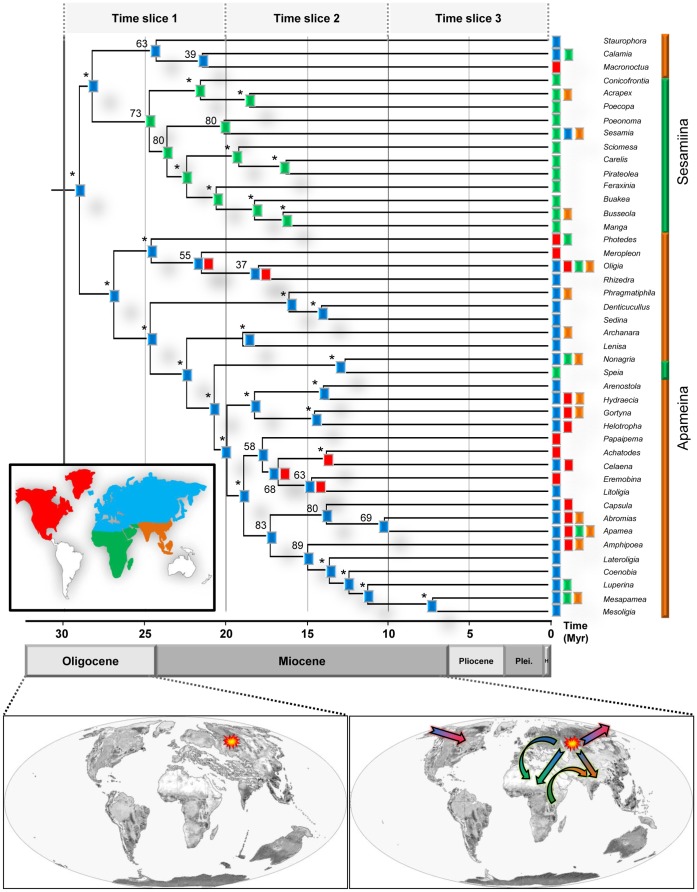
Historical biogeography of the tribe Apameini reconstructed using the DEC model based on the median-BEAST chronogram. A coloured square indicating the most likely ancestral area is displayed at each node of the topology. Colours of the squares correspond to the colour of the areas presented in the map at the bottom left corner of the chronogram. Asterisks above the nodes indicate a strong support for the recovered areas (>2 log-likelihood units against the second better score). A 5 Myr-timescale is placed at the bottom of the chronogram and goes from the mid-Oligocene to the Holocene. The vertical bars next to the genera names delimit the two subtribes (Apameina in orange and Sesamiina in green). The biogeographical scenario for the tribe is presented in two maps at the bottom of the figure. Dotted-lines above the maps specify the related epoch (Left map: Oligocene and right map: Miocene). The origin of the group is highlighted by a yellow and red symbol, and the major dispersal events are shown with arrows.

Following the principles described in Ree and Smith [63], we constructed temporal constraints on rates of dispersal between the possible character states. These constraints were implemented as a series of three time slices, but only for the biogeographic analyses. No specific constraint on transition rates between host-plant groups was imposed for the host-plant analyses, since no relevant information was available to set specific rates (all transition rates were thus set to to 1.0). All time slices together spanned the past 30 Myr, with each slice being 10 Myr in duration. For each time slice, we constructed a matrix of scaling factors (between zero and 1) for the dispersal rate between areas according to their temporal position [66], interpreting greater origins as being inversely proportional to the expected rate. Therefore, three models were constructed: (*i*) a null model M0 in which all the dispersal rates between areas were set to a fixed value of 1.0 for every time slice; (*ii*) a M1 model in which the values of dispersal rates were fixed according to the geological pattern of each period with 0.1 being the dispersal rate between Afrotropics and Nearctic; and (*iii*) a M2 model in which the values are similar to the M1 model except for the dispersal rate between Afrotropics and Nearctic, being fixed at 0.01 in order to investigate the biogeographical pattern with a LDD-like (long-distance dispersal) model (see Table S2 in the electronic supplementary materials for the matrix of dispersal constraints).

Supplementary analyses were further conducted to determine the best-fit ancestral root area and the ancestral set of host-plants, using local optimizations conditional on the root state. These optimizations were conducted for all models and considering single and multiple character states. A 2 log-likelihood unit’s threshold was used to choose which area or set of host-plants was better supported [63].

**Table 5:** Model-based analysis of diversification rates in Apameini moths.

Models	Constant-Rate		Varying-Rate		Decreasing-Rate	
	log *L*	AIC (ÄAIC^a^)	log *L*	AIC (ÄAIC^a^)	log *L*	AIC (ÄAIC^a^)
å = 0	−262.39	526.77 (29.59)^b^	−245.59	497.18 (0)	−255.39	516.77 (19.57)
NDR c	r = 0.207		r1 = 0.17/r2 = 0.327		r1 = 0.214/r2 = 0.052	
å = 0.35*	−261.89	525.78 (28.6)^b^	−248.04	502.08 (4.89)	−255.3	516.6 (19.42)
NDR c	r = 0.179		r1 = 0.146/r2 = 0.289		r1 = 0.187/r2 = 0.039	
å = 0.5	−262.09	526.18 (28.99)^b^	−249.78	506.59 (8.38)	−255.71	517.42 (20.24)
NDR c	r = 0.164		r1 = 0.133/r2 = 0.266		r1 = 0.171/r2 = 0.032	
å = 0.95	−274.78	551.56 (54.38)	−268.96	543.93 (46.74)	−269.49	544.99 (47.81)
NDR c	r = 0.049		r1 = 0.035/r2 = 0.09		r1 = 0.052/r2 = 0.004	

Varying-rate model considers all possible bipartitions of tree and finds bipartition giving the highest likelihood when net diversification rates are optimized separately to each partition. Decreasing-rate model assumes that previously recovered (with varying-rate model) shift point node have retained the ancestral diversification rate present at the root node.

a ΔAIC is the difference in AIC scores between each model and the overall best-fit model (here, varying-rate model with ε = 0).

b The data reject the constant-rate model in favour of the varying-rate model under ε = 0 and 0.95 (*p*<0.001). Rate-decrease model does not show a simple nested relationship with varying-rate model, but AIC strongly favours the varying-rate model.

c Maximum-likelihood estimate of the net diversification rate r in lineages Myr^−1^ (r, r1 and r2 are the net diversification rate of the subtree partition containing the nine Apameina genera where diversification rates shifted).

* Maximum likelihood estimate of the extinction rates ε in lineages Myr^−1^.

### Diversification Rates

To investigate the diversification rates over time, we followed a step-by-step procedure using the program R 2.14 with the *ape* [67], *laser* [68], and *geiger* packages [69]. Macro-evolutionary analyses were realized on the genus-level tree created by pruning all non-Apameini genera from the BEAST chronogram. The species richness was assigned to each genus, following the data in the literature [9], [11], [12], [14], [17], [38], using the function *getTipdata*. Since the taxon sampling is not complete for all Apameini genera, it is difficult to distinguish between crown- and stem-group ages [5], [70]. Consequently, in a conservative way we only used the stem age of each genus for all corresponding analyses.

First, we plotted the number of species within the different Apameini genera as a function of stem clade age, as inferred from the BEAST chronogram, in order to see possible relationships between clade age and diversity [3] (see Figure S3 in the electronic supplementary materials). Then, we tested whether specific lineages of Apameini are characterized by exceptionally high or low net diversification rates (speciation minus extinction) using the estimator of Magallón and Sanderson [5]. Specific diversification rates were estimated for each genus and for the two lineages encompassing the predominantly Holarctic Apameina and Afrotropical Sesamiina. Therefore, all corresponding estimations of diversification rates were carried out using a birth-death model, given the stem age and extant diversity, with three distinct relative extinction rates (ε = 0/0.5/0.95).

Second, we tested for shifts in diversification rates within the Apameini using a similar approach to MEDUSA [70], [71]. This method compares the likelihood of the data fitting a model with constant diversification rates for all lineages (constant-rate) with the likelihoods under two varying-rate models where an ancestral diversification rate *r1* shifts to a new rate *r2* along some branches in the tree (varying-rate; [32], [70]). The first varying-rate model allows change (increase or decrease) in diversifications rates (from a putative ancestral rate *r1* to a new rate *r2*) along some branches in the tree. The second varying-rate model constrains a rate-decrease scenario between the root node and the tips. These two models allow testing for two competing hypotheses such as: (*i*) species-rich genera would show an increase in diversification rates relative to species-poor lineages, or (*ii*) these specious genera retained, in fact, an ancestral but elevated diversification rate, while other genera exhibited a decline in diversification [72]. For these comparisons, we used a birth-death estimator based on both phylogenetic and taxonomic data (known species richness of each genus) following the approach of Rabosky *et al.* ([70]; see also [71]). All resulting likelihood scores were compared through likelihood ratio tests and AIC to determine the best-fit model for the data.

Interestingly, this approach also allows detecting the location of shift points, which are associated with sudden increases in likelihood scores [71]. The first shift point is the maximum likelihood score. Because of the possible occurrence of multiple shifts in diversification rates, a threshold in likelihood scores was used [70]. To avoid conditioning results on any particular tree topology and branch lengths, 1,000 random time-calibrated phylogenies were sampled from the post-burn-in tree of the BEAST analysis. All analyses were repeated based on this set of trees to generate the posterior distribution of likelihood differences between all diversification models.

## Results

### Molecular Phylogeny

The final molecular matrix comprises 94 species representative of 92 genera (in which 44 are Apameini genera) and 1,905 bp. The Bayes factors for each partitioning strategy are listed in Table 1 and 2 (see Table S3 in the electronic supplementary materials for the best-fit evolutionary models selected for each partition under the BIC criterion). The strategy with two partitions (first and second positions against the third) was selected for both the COI and EF-1α genes, whereas the most complex strategy encompassing six different partitions (a partition for each coding position of the two genes) was selected for the combined dataset. The effective sample size (ESS) for all the parameters of the independent runs were higher than 500 except for the strategy (*NoPart*) of the combined dataset [48].

As expected, the gene trees of EF-1α and COI yielded different topologies in accordance with their nature (nuclear vs. mitochondrial). If the topology based on EF-1α sequences presents a moderate or good support for most of the deepest nodes and recovers the main groups monophyletic, on the other hand, the COI topology presents weak support for the basal nodes but robust ones for the nodes of closely-related genera. However, the phylogenetic signals for the two genes revealed no significantly supported incongruence (Figure S1 and S2 for the best topology of each gene). The phylogenetic hypothesis highlighted by the MrBayes analyses of the combined dataset yielded well-resolved clades with good supports for the major nodes of the topology (Figure 1). The superfamily Noctuoidea along with the families Erebidae (PP = 0.97), Euteliidae (PP = 0.74) and Nolidae (PP = 0.83) are found monophyletic as well as the family Noctuidae despite a lower support (PP = 0.63). The tribe Apameini (node N1 in Figure 1) is found monophyletic with a PP of 0.96. The Afrotropical clade of the subtribe Sesamiina is found paraphyletic because of the placement of the genus *Speia*. The clade (N2) corresponding to the subtribe without this genus is found monophyletic with a strong support (PP = 0.95). The Holarctic clade of the subtribe Apameina (N3) is also found to be paraphyletic because of the placement of the genera *Staurophora* and *Calamia* and of the Nearctic genus *Macronoctua*. However, the support for the clade containing the rest of the Apameina genera with the genus *Speia* is weak (PP = 0.20) along with the support for the clade containing the node N2 and the three genera before-mentioned, indicating a lack of phylogenetic signal and a need for more data. In addition to these results, the tribe Apameini is recovered within the subfamily Cuculiinae (PP = 0.97) represented by the genera *Anathix*, *Aporophyla* and *Litholomia* in the data sampling.

### Divergence Times

Over the eight calibration sets analysed, the one comprising a Yule model of speciation, no constraint based on the age of the Hawaiian archipelago and no correction on the constraint ages was selected according to the BF recovered in Tracer (Table 3). The chronogram (Figure 2) obtained through the Bayesian dating analyses allows us to infer a median age of 29.08 Myr for the Apameini clade (node N1), with 95% highest posterior densities (HPD) of 20.96–42.76 Myr (Table 4). Beyond this origin coeval with the mid Oligocene, the subtribe Sesamiina excluding the genus *Speia* (node N2) began to diversify around 24.73 Myr (95% HPD: 17.75–36.60 Myr), then radiated between the Oligocene-Miocene transition and the early Miocene. The subtribe Apameina (node N3) with the genus *Speia* and excluding the three genera *Staurophora*, *Calamia* and *Litholomia* presents the same pattern of evolution with a mean age of 26.93 Myr (95% HPD: 19.18–39.66 Myr) and a specific radiation initiated during the late Oligocene. Eventually, even if the molecular sampling is depleted regarding the extreme diversity of the main groups presented in this study, our dating analyses also provided respective median ages for the Noctuoidea super-family (84.34 Myr, 95% HPD: 62.11–123.54 Myr) and the Noctuidae (60.33 Myr, 95% HPD: 45.47–88.15 Myr) and Erebidae (55.49 Myr, 95% HPD: 45.96–79.94 Myr) families, which may represent a first step for further investigations.

### Reconstruction of Host-plant Associations

The main results of the analyses carried out under the program Lagrange are presented in Figure 3. The family Poaceae is statistically recovered (>2 log-likelihood units against the second best score) as the most likely ancestral host plant group for the root within the unconstrained model (no prior for the root) (see Table S4 in the electronic supplementary materials for the likelihood scores at the root). Results of the optimization with Poaceae being inferred at the root are presented in Figure 3. Most of the nodes statistically recover the family Poaceae as optimal host plant group. The only three nodes that recover different characters are terminal and present the same ancestral character state as their current one (i.e., both *Nonagria* and *Speia* are exclusively indentured to the family Typhaceae, and the reconstructed character for their corresponding node is Typhaceae). The shifts from the family Poaceae to different families are thus mostly Miocenic in age or even more recent for some genera. These results support an initial specialization of the tribe Apameini over the family Poaceae, contemporary with the period between the emergence of grasses in the late Oligocene and their dominance in the early Miocene.

### Historical Biogeography

The models M0, M1 and M2 yielded the same results for all the nodes and only differ by their respective likelihoods. The model M1 was recovered with a higher likelihood and was thus selected (see Table S5 in the electronic supplementary materials for the likelihood scores at the root). The reconstruction of ancestral areas for each node of the calibrated topology is shown in Figure 4. Under the unconstrained model, the Palaearctic region is recovered as the most likely ancestral area for the root with significant difference in likelihood from the other putative areas (ln *L* = −62.9). The main biogeographical pattern highlighted by the DEC analysis supports one major dispersal event within the African continent around 25 Myr ago, with more recent colonization events from the Palaearctic region to the Nearctic region in different lineages of the tree.

### Diversification Rates

First, the net diversification rates of Palaearctic species and Afrotropical species were estimated at 0.229/0.217/0.163 per Myr and 0.176/0.165/0.114 per Myr respectively, using a birth-death model and three extinction rates (ε = 0/0.5/0.95). These respective diversification rates are significantly different under likelihood ratio tests (one-tailed test; *p*<0.001). Estimates of net diversification rates for each genus are given in Figure 3. These results indicate that some genera are characterized by low diversification rates (e.g., *Staurophora*) whereas other genera experienced high and even exceptional (e.g., *Apamea*) diversification rates.

Second, comparisons of observed species richness within genera relative to those expected under a homogeneous net diversification rate across all Apameini moths reject the null hypothesis that net diversification rates have remained constant among lineages (for ε = 0: ΔAIC = 29.59, *p*<0.001; for ε = 0.5: ΔAIC = 19.59, *p*<0.001; for ε = 0.95: ΔAIC = 7.63, *p* = 0.002; see Table 5 for details). Regardless of whether Apameini diversification rates are estimated using the combined taxonomic/phylogenetic approach, there is clearly an excess of species-poor or species-rich lineages (Figure 3). Although this test is conservative [69], these results are robust to assumptions about the underlying model of extinction (similar results with ε = 0/0.5/0.95). Therefore, clade age alone does not explain the striking disparity in species richness among Apameini moths (see Figure S3 in the electronic supplementary materials).

The data also clearly reject the constant diversification model in favor of varying-rate models (*p*<0.001). The varying rate model specifying increased diversification outperforms the second varying-rate model (with a rate-decrease scenario), as assessed by the AIC and likelihood scores. This result is not conditional on the topology and branch lengths shown in Figure 2, because the difference in likelihood scores between the (increased diversification) varying-rate and decreasing-rate models strongly favors the former model (*p*<0.001). Based on the best-fit varying-rate model, the ML estimate of the shift point is the node corresponding to the common ancestor of a large clade including nine genera of Apameina grouping together 227 species (Figure 3). This node is dated around 17.5 Myr ago and fits well with a major Cenozoic climate change, the middle Miocene climatic optimum [19].

Palaeontological records support the fact that relative extinction rates have generally been high, with no evidence suggesting that extant clades have diversified in the absence of extinction [72]. It is thus probable that the true magnitude of the rate increase for the node exceeded that inferred under ε = 0. When estimating the relative extinction rate, diversification analyses estimated an ML for ε = 0.35 at −261.89 under a constant-rate model and at −248.04 under a varying-rate model (Table 5). Under extinction rates of both ε = 0 and 0.5, ML estimates of net diversification rates for this node and all other Apameini lineages suggest that the net diversification rate within this clade has increased approximately 2-fold relative to that observed across the remainder of the tree (ε = 0/0.35/0.5).

Rate shifts at other internal nodes are far less likely than those at the node for the nine Apameina genera, as assessed by the difference in likelihood scores (Δ*L*) between the best-fit location of the rate shift and alternative nodes. However, a single node has a close likelihood: the common ancestor of the genera *Abromias* and *Apamea* (Δ*L* = 2.47, *p* = 0.085 for ε = 0 but results for other values of ε are qualitatively similar). This node is comprised within the clade of the nine genera where the first shift occurred (Figure 3).

## Discussion

### Origin and Early Diversification of the Apameini

According to our results, the currently worldwide-distributed moths of the Apameini tribe may have originated around 29 Myr ago in the Palaearctic region even if no specimen of the Palaearctic subtribe Oxytripiina was included in the dataset. Concerning the latter, we suggest that the inclusion of this subtribe would be of minor impact for the ancestral character reconstruction process since the two species of this group are merely West Palaearctic. During the Oligocene, the Earth was experiencing deep changes especially through a major climate turnover [19]. The global cooling, initiated by the Terminal Eocene Event 34 Myr ago, has facilitated the establishment of a completely different set of biomes for which species were not necessarily adapted [30], [73], [74]. In particular, the depletion of atmospheric CO2 values associated with a growing aridity begets the transition from C_3_ to C_4_ species, favouring the emergence of new ecological niches [21]. Already existing but less diversified monocot families such as Poaceae, Cyperaceae or Liliaceae [75] took advantage of the major turnovers to spread and dominate ecosystems such as grasslands, savannahs and remaining wet ecotones [21], [23], whereas the tropical forests dispersed southwards [76]. It appears that the ancestor of the Apameini was a specialist on the family Poaceae (Figure 3), and benefited from the grasslands dominance that radiated around 31 Myr ago [57] and spread in the middle Miocene [18], [20]–[22], [25], [26].

### Historical Biogeography

From the crown of the tribe, ancestors have evolved in two main clades chiefly matching the extant temperate Apameina and tropical Sesamiina (Figure 4). Dating analyses indicate that these ecological groups originated during the late Oligocene and are equal in age. From the Palaearctic region in which the tribe have seemingly originated, ancestor of Sesamiina dispersed in Afrotropics 25 Myr ago. The rest of the major clade from the Apameini diversified in the Palaearctic region since 27 Myr to give the majority of the extant Apameina genera. Interestingly, it appears that despite being numerous and spread in the whole topology, Oriental species seem to be more recent. This pattern may be the result of recent dispersals from Afrotropics towards Oriental region, along with a combination of climatic and geological factors that could have broken the colonization of Oriental region from Palaearctic. However, regarding the lack of Oriental genera in our sampling, the apparent youth of Oriental lineages might be artefactual.

Several studies have highlighted divergent conclusions regarding the geographical origin of the grasslands between the late Palaeogene and the early Neogene. However it seems, according to carbon isotopes and fossil evidences that the apparition of the first grass-dominated habitats might have taken place in the New World and especially the Neotropical region [21], [77], [78]. Independently, the Palaearctic and Oriental regions are thought to be the origin of the Old World ancestral grassland landscapes, allowing in second place the colonization of Afrotropics, then Australian biomes [21], [77], [79], [80]. This complex Old World evolutionary pattern is congruent with the biogeographical history inferred from our biogeographic results, in which the migration and evolution of the Apameini tribe ancestors could have been deeply influenced not only by the climatic change initiated at this time, but also by the progressive spread of their host-plants. Our results suggest more recent and multiple independent colonization of the Nearctic region during the Miocene across the phylogeny (Figure 4). Such a dispersal event could have been facilitated by the presence of the Beringian Bridge II from the mid-late Miocene to the late Pliocene [81]. At that time, a wide spit of coniferous forestland extended from the North-western Nearctic to the North-eastern Palaearctic and less likely by the Thulean land bridge constituted by an island chain between West Palaearctic and East Nearctic, thought to have existed until the late Oligocene [81]. Interestingly, the recovery of the Nearctic region as one of the most recent colonized part of the World might explain the absence of Apameini representatives in the Neotropics.

### Tempo and Mode of Diversification

The diversification patterns observed within Satyrini butterflies, Anisopliina beetles and Cicadellidae leafhoppers suggest not only a predominant role of grasslands apparition around the Oligocene and Miocene boundary, but also the contribution of abiotic factors such as biogeographic and climatic events, driving the diversification during the late Miocene [31]–[33]. However, those studies highlight a diversification pattern of phytophagous insects originated around the Oligocene, whereas the tribe Apameini seems to have diversified later in the early Miocene, suggesting a close association between the apparition and dominance of their host-plants and the major climatic turnover of the middle Cenozoic as well (Figures 2, 3, 4). Our age estimates indicate that the diversifications of Apameini and their grasslands seem to have occurred in the same timeframe, between the early and the middle Miocene [18], [21], [57]. The high level of host-plant specialization within the Apameini moths, namely endophagy and enlargement in mandibular muscles in larvae, correlated with the brisk radiation underlined by our results, suggests an evolutionary pattern similar to the one spotlighted within the mammals [18], [20], [27].

Contrary to numerous studies focused on the diversification of groups during the Miocene, we have conducted ML diversification analyses [70], [71] that indicate diversification rates have not remained constant through time for this group. Most importantly, we were able to locate the shift point in diversification rates, which corresponds to a species-rich clade that originated in the Palaearctic region during the early Miocene. Contrary to numerous studies that argued for slowdowns in diversification rates along the evolutionary history [82], we evidence a 2-fold increased diversification rates after the shift. It suggests that this group of specialist grass feeders (Poaceae, see Figure 3) experienced high or explosive net diversification rates, similar to other groups of organisms that also diversified in the middle Miocene (e.g., Australian lizards [69]; Parnassiinae [59]; Proteaceae [83]). Interestingly, this period precisely corresponds to a period when open and grassy habitats became more dominant [20], [23]. Such diversification pattern may be attributed to adaptive radiation of these moths with explosive diversification rates after the appearance of these biomes. Thus, our molecular dating and diversification analyses likely support the hypothesis that the radiation of Apameini grass feeders is connected to the transition to open grasslands in the Miocene.

Because our analyses are partially dependent on taxonomy, our results could have been influenced by a failure to adequately account for true species richness within each genus. Up to our knowledge, few interspecific and intraspecific phylogenetic studies have been conducted on Apameini moths, even if Zilli and collaborators have already began a full revision of the tribe which will be of precious value for future investigations [10]. From our knowledge, it seems that at least the subtribe Sesamiina could possibly contain a high level of cryptic diversity [84]. However, this issue would only influence our results if the nine Apameina genera harbour proportionately fewer cryptic species than other taxa.

### Conclusions

This study constitutes the first attempt to our knowledge, to untangle the origins and evolution of the Apameini moths to date, and brings additional contribution to the study of evolutionary patterns within phytophagous insects in relation with the impact of climate-linked environmental changes. The evolution of the group was likely shaped by a combination of biogeographic (range expansion, dispersal event), and ecological (host-plant specialization), as well as climatic (Miocene cooling) processes which played an important role in the colonization of more open and arid habitats, in relation with late Cenozoic changes. Here, we infer that the major turnover in plant communities might have shaped an adaptive radiation between the Apameini moths and their host-plants during the Miocene. However, a denser taxon sampling and additional analyses of molecular species delimitation could enable us to gain new insights in the close association between palaeoenvironmental changes and diversification of those specious moths.

## Supporting Information

Figure S1 **COI tree of the tribe Apameini within the superfamily Noctuoidea under Bayesian inference.** Posterior probabilities (PP) are indicated above the nodes.
(TIF)

Figure S2 **EF-1α tree of the tribe Apameini within the superfamily Noctuoidea under Bayesian inference.** Posterior probabilities (PP) are indicated above the nodes.
(TIF)

Figure S3 **Relationship between stem clade age and extant diversity for Apameini moths.** Results indicate that there is no evident relationship between the age of a group and its diversity. *Apamea* and *Abromias* are recent lineages and species-rich, and many remaining lineages have a deficit of species. It appears that variations in diversification rates among lineages explain the disparity in biodiversity among the Apameini genera. Net diversification rate was estimated from combined taxonomic and phylogenetic data.
(TIF)

Table S1 **Taxon sampling and GenBank accession numbers for the sequences used in the molecular matrix (when ‘-’ is indicated, the gene was not successfully sequenced or retrieved).**
(DOCX)

Table S2 **Transitional matrices used for the biogeographic analyses performed with Lagrange and the DEC model.** A, Afrotropics; B, Nearctic; C, Palaearctic; and D, Oriental. M1 is the model with long-distance dispersal set to 0.1 and M2 has the long-distance dispersal set to 0.01 (M0 has equal transitional rates for all areas, i.e., null model).
(DOCX)

Table S3 **Selection of the best substitution models for each partition under jModelTest using AIC and BIC criterions.**
(DOCX)

Table S4 **Reconstruction of ancestral host plant characters for Apameini using Lagrange.** A, Amaryllidaceae; B, Cyperaceae; C, Iridaceae; D, Juncaceae; E, Liliaceae; F, Poaceae; G, Typhaceae; and H, Dicotyledons. The Poaceae are the ancestral host plant of Apameini.
(DOCX)

Table S5 **Reconstruction of ancestral area for Apameini using Lagrange.** A, Afrotropics; B, Nearctic; C, Palaearctic; and D, Oriental. The Palaearctic region is the ancestral area of Apameini for the three models.
(DOCX)
