# Supplementary figures and images for: Palaeoenvironmental Shifts Drove the Adaptive Radiation of a Noctuid Stemborer Tribe (Lepidoptera, Noctuidae, Apameini) in the Miocene
Source: PLoS One. 2012 Jul 31;7(7):e41377. doi: 10.1371/journal.pone.0041377 (PMC3409182; doi:10.1371/journal.pone.0041377)

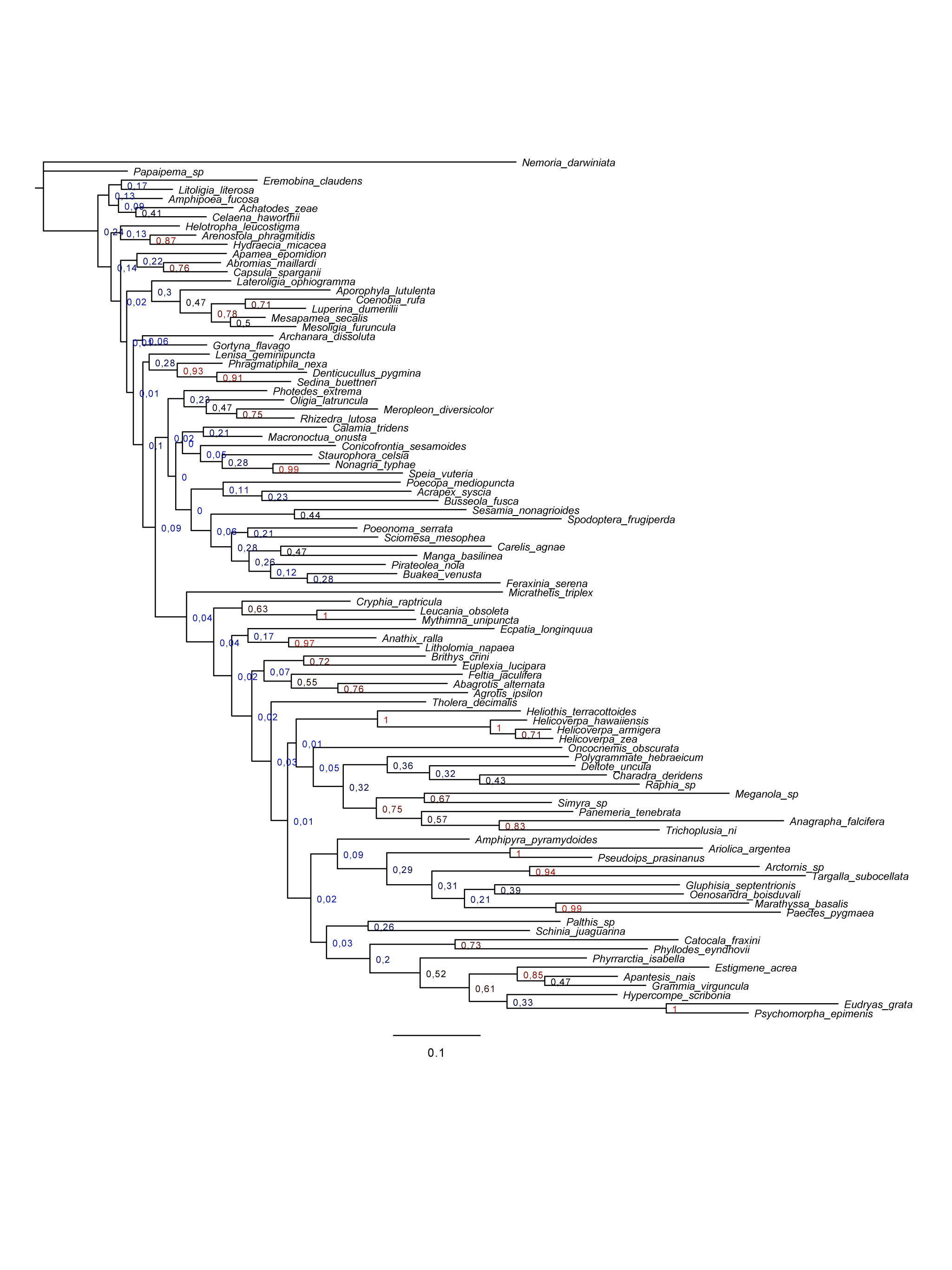

Supplement: Figure S1 — COI tree of the tribe Apameini within the superfamily Noctuoidea under Bayesian inference. Posterior probabilities (PP) are indicated above the nodes. (TIF) [file pone.0041377.s001.tif]

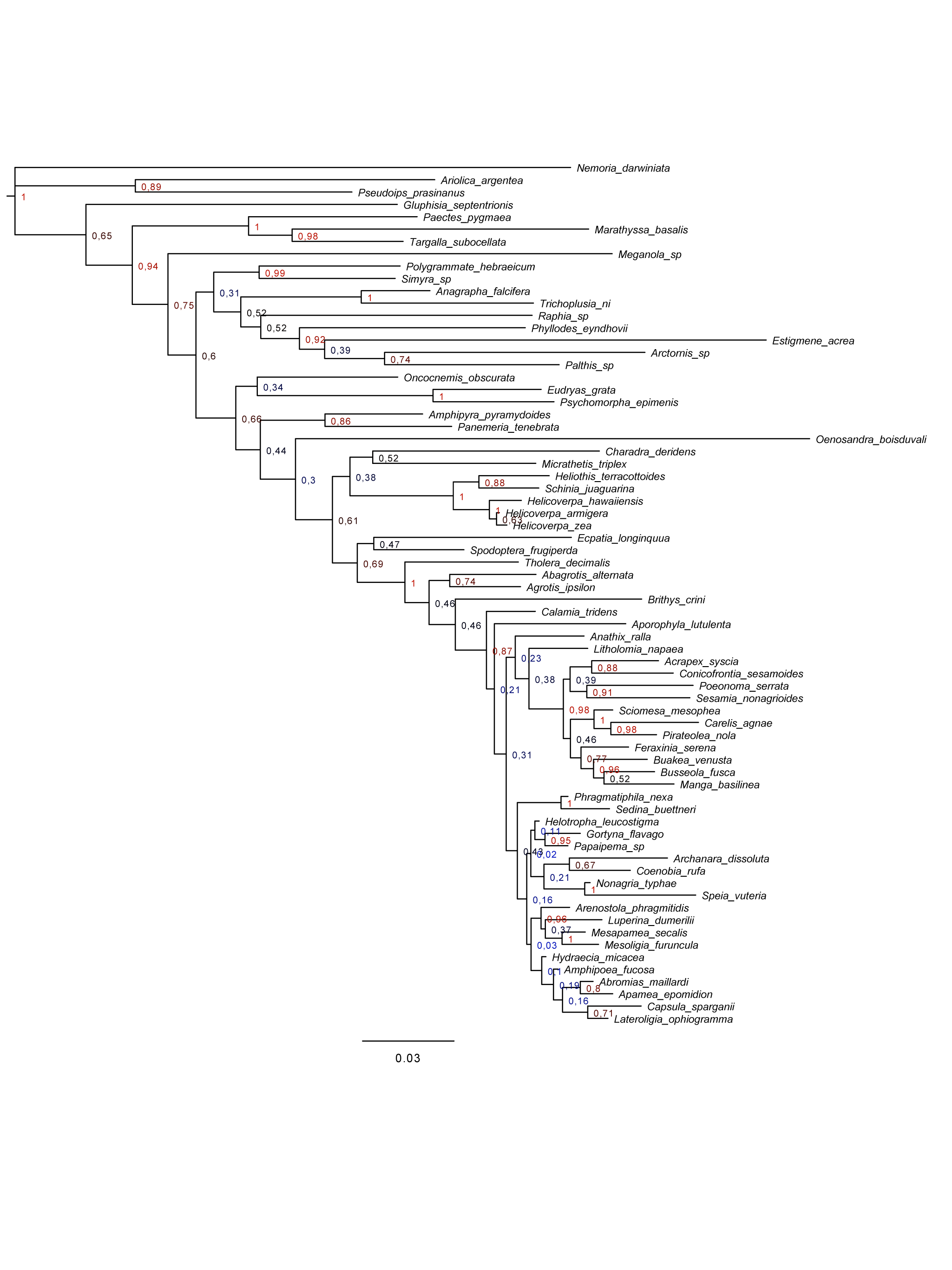

Supplement: Figure S2 — EF-1α tree of the tribe Apameini within the superfamily Noctuoidea under Bayesian inference. Posterior probabilities (PP) are indicated above the nodes. (TIF) [file pone.0041377.s002.tif]

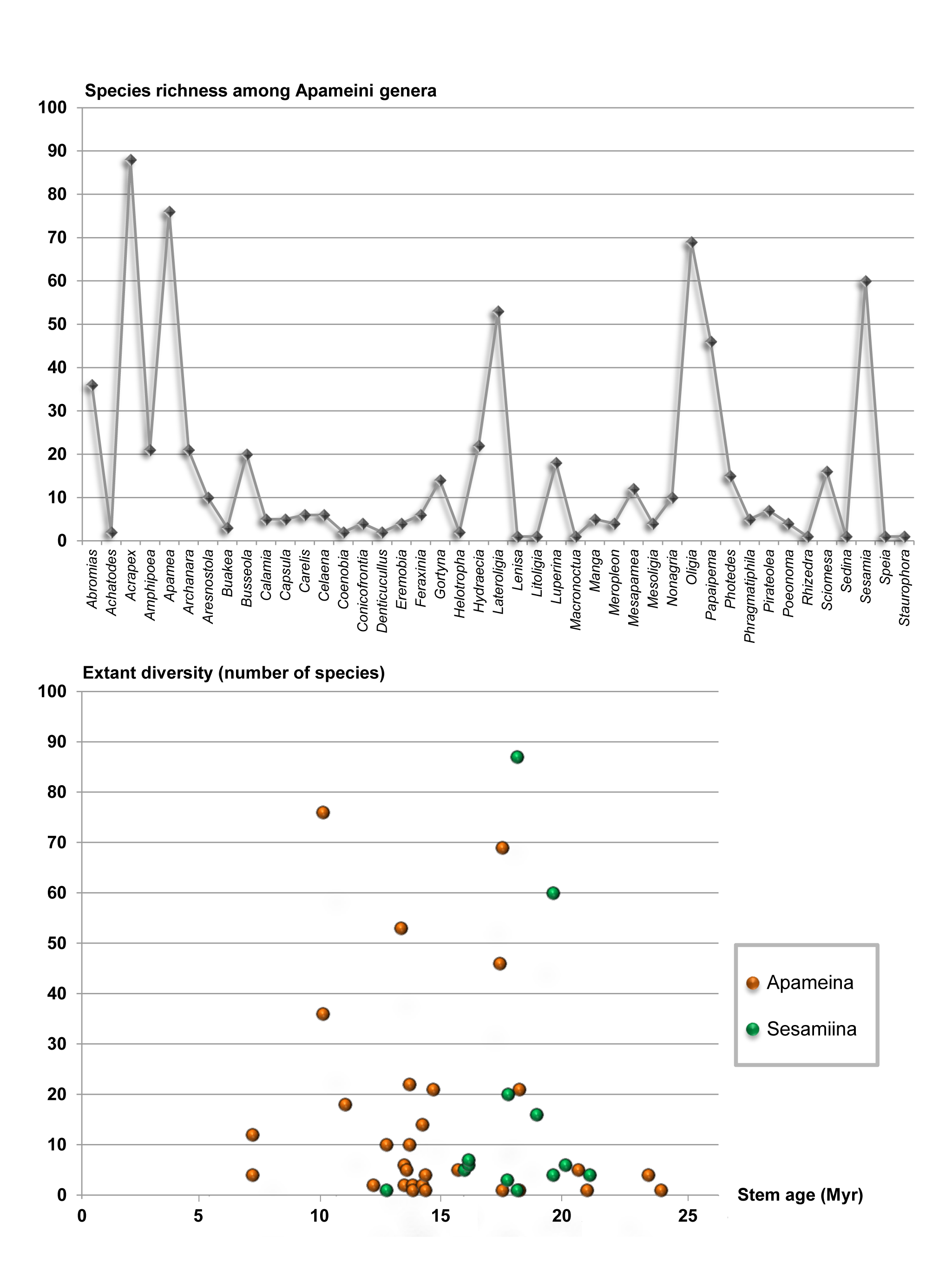

Supplement: Figure S3 — Relationship between stem clade age and extant diversity for Apameini moths. Results indicate that there is no evident relationship between the age of a group and its diversity. Apamea and Abromias are recent lineages and species-rich, and many remaining lineages have a deficit of species. It appears that variations in diversification rates among lineages explain the disparity in biodiversity among the Apameini genera. Net diversification rate was estimated from combined taxonomic and phylogenetic data. (TIF) [file pone.0041377.s003.tif]
