# Supplementary material for: Palaeoenvironmental Shifts Drove the Adaptive Radiation of a Noctuid Stemborer Tribe (Lepidoptera, Noctuidae, Apameini) in the Miocene
Source: PLoS One. 2012 Jul 31;7(7):e41377. doi: 10.1371/journal.pone.0041377 (PMC3409182; doi:10.1371/journal.pone.0041377)
Supplement: Table S1 — Taxon sampling and GenBank accession numbers for the sequences used in the molecular matrix (when ‘-’ is indicated, the gene was not successfully sequenced or retrieved). (DOCX) [file pone.0041377.s004.docx]

**Table S1.** Taxon sampling and GenBank accession numbers for the sequences used in the molecular matrix (when ‘-‘ is indicated, the gene was not successfully sequenced or retrieved).

| Species name | Cytochrome oxidase subunit 1 | Elongation factor 1 alpha |
| --- | --- | --- |
| *Abagrotis alternata* | GU089561 | AF151630 |
| *Abromias maillardi* | JX282423 | JX282466 |
| *Achatodes zeae* | GU089570 | - |
| *Acrapex syscia* | JX282424 | JX282467 |
| *Agrotis ipsilon* | GU686965 | U85704 |
| *Amphipoea fucosa* | JX282425 | JX282468 |
| *Amphipyra pyramydoides* | HQ964488 | U85693 |
| *Anagrapha falcifera* | HM867238 | U85686 |
| *Anathix ralla* | EU768892 | U85702 |
| *Apamea epomidion* | JX282426 | JX282469 |
| *Apantesis nais* | GU089361 | EU333575 |
| *Aporophyla lutulenta* | JX282427 | JX282470 |
| *Archanara dissoluta* | JX282428 | JX282471 |
| *Arctornis sp* | JF415316 | HQ006335 |
| *Arenostola phragmitidis* | JX282429 | JX282472 |
| *Ariolica argentea* | HQ006937 | HQ006421 |
| *Brithys crini* | JX282430 | JX282473 |
| *Buakea venusta* | JX282431 | JX282474 |
| *Busseola fusca* | JX282432 | JX282475 |
| *Calamia tridens* | JX282433 | JX282476 |
| *Capsula sparganii* | JX282434 | JX282477 |
| *Carelis agnae* | JX282435 | JX282478 |
| *Catocala fraxini* | GU707317 | FJ768771 |
| *Celaena haworthii* | HQ563355 | - |
| *Charadra deridens* | GU679205 | U85683 |
| *Coenobia rufa* | JX282436 | JX282479 |
| *Conicofrontia sesamoides* | JX282437 | JX282480 |
| *Cryphia raptricula* | HQ565501 | GU829320 |
| *Deltote uncula* | JF415772 | GU829315 |
| *Denticucullus pygmina* | JX282438 | JX282481 |
| *Ecpatia longiquua* | HQ006894 | HQ006380 |
| *Eremobina claudens* | JX282439 | - |
| *Estigmene acrea* | GU091095 | EU333578 |
| *Eudryas grata* | GU087808 | U85697 |
| *Euplexia lucipara* | JX282440 | - |
| *Feltia jaculifera* | HQ964376 | AF173390 |
| *Feraxinia serena* | JX282441 | JX282482 |
| *Gluphisia septentrionis* | HM870241 | AF151603 |
| *Gortyna flavago* | JX282442 | JX282483 |
| *Grammia virguncula* | EU119535 | EU333582 |
| *Helicoverpa armigera* | GQ892855 | FJ768770 |
| *Helicoverpa hawaiiensis* | EU768939 | EU769063 |
| *Helicoverpa zea* | GU438926 | U20136 |
| *Heliothis terracottoides* | EU768926 | AF151631 |
| *Helotropha leucostigma* | JX282443 | JX282484 |
| *Hydraecia micacea* | JX282444 | JX282485 |
| *Hypercompe scribonia* | GU087871 | EU333588 |
| *Lateroligia ophiogramma* | JX282445 | JX282486 |
| *Lenisa geminipuncta* | JX282446 | JX282487 |
| *Leucania obsoleta* | JX282447 | - |
| *Litholomia napaea* | HM862893 | AY952637 |
| *Litoligia literosa* | JF860032 | - |
| *Luperina dumerilii* | JX282448 | JX282488 |
| *Macronoctua onusta* | GU438990.1 | - |
| *Manga basilinea* | JX282449 | JX282489 |
| *Marathyssa basalis* | HQ006887 | HQ006374 |
| *Meganola sp* | JN305219 | U85675 |
| *Meropleon diversicolor* | GU439013 | - |
| *Mesapamea secalis* | JX282450 | JX282490 |
| *Mesoligia furuncula* | JX282451 | JX282491 |
| *Micrathetis triplex* | GU163423 | AY952602 |
| *Mythimna unipuncta* | JX282452 | AF151627 |
| *Nemoria darwiniata* | HM869695 | EU151683 |
| *Nonagria typhae* | JX282453 | JX282492 |
| *Oenosandra boisduvali* | GU929762 | GU829377 |
| *Oligia latruncula* | JX282454 | - |
| *Oncocnemis obscurata* | EU779854 | U85685 |
| *Paectes pygmaea* | GU090070 | U85674 |
| *Palthis sp* | JN304888 | U85678 |
| *Panemeria tenebrata* | HQ563401 | HQ006349 |
| *Papaipema sp* | HQ964356 | AF151628 |
| *Photedes extrema* | JX282455 | - |
| *Phragmatiphila nexa* | JX282456 | JX282493 |
| *Phyllodes eyndhovii* | HQ006931 | HQ006415 |
| *Phyrrarctia isabella* | GU094281 | EU333593 |
| *Pirateolea nola* | JX282457 | JX282494 |
| *Poecopa mediopuncta* | JX282458 | JX282495 |
| *Poeonoma serrata* | JX282459 | JX282496 |
| *Polygrammate hebraeicum* | GU087511 | U85688 |
| *Pseudoips prasinanus* | JF860127 | GU829229 |
| *Psychomorpha epimenis* | EU779855 | U85691 |
| *Raphia sp* | HM867664 | U85689 |
| *Rhizedra lutosa* | JX282460 | JX282497 |
| *Schinia jaguarina* | EU768958 | EU769075 |
| *Sciomesa mesophea* | JX282461 | JX282498 |
| *Sedina buettneri* | JX282462 | JX282499 |
| *Sesamia nonagrioides* | JX282463 | JX282500 |
| *Simyra sp* | GU091422 | AY952601 |
| *Speia vuteria* | JX282464 | JX282501 |
| *Spodoptera frugiperda* | HQ964487 | U20139 |
| *Staurophora celsia* | JF415659 | - |
| *Targalla subocellata* | HQ006914 | HQ006397 |
| *Tholera decimalis* | JX282465 | JX282502 |
| *Trichoplusia ni* | GU439174 | U20140 |
