# Supplementary material for: Palaeoenvironmental Shifts Drove the Adaptive Radiation of a Noctuid Stemborer Tribe (Lepidoptera, Noctuidae, Apameini) in the Miocene
Source: PLoS One. 2012 Jul 31;7(7):e41377. doi: 10.1371/journal.pone.0041377 (PMC3409182; doi:10.1371/journal.pone.0041377)
Supplement: Table S2 — Transitional matrices used for the biogeographic analyses performed with Lagrange and the DEC model. A, Afrotropics; B, Nearctic; C, Palaearctic; and D, Oriental. M1 is the model with long-distance dispersal set to 0.1 and M2 has the long-distance dispersal set to 0.01 (M0 has equal transitional rates for all areas, i.e., null model). (DOCX) [file pone.0041377.s005.docx]

**Table S2.** Transitional matrices used for the biogeographic analyses performed with Lagrange and the DEC model.

A, Afrotropics; B, Nearctic; C, Palearctic; and D, Oriental. M1 is the model with long-distance dispersal set to 0.1 and M2 has the long-distance dispersal set to 0.01 (M0 has equal transitional rates for all areas, i.e., null model).

| **0-10 Myr** | A | B | C | D |
| --- | --- | --- | --- | --- |
| A | - | 0.1 (M1)/0.01 (M2) | 1.0 | 0.75 |
| B | 0.1 (M1)/0.01 (M2) | - | 0.1 (M1)/0.01 (M2) | 0.1 (M1)/0.01 (M2) |
| C | 1.0 | 0.1 (M1)/0.01 (M2) | - | 1.0 |
| D | 0.75 | 0.1 (M1)/0.01 (M2) | 1.0 | - |

| **10-20 Myr** | A | B | C | D |
| --- | --- | --- | --- | --- |
| A | - | 0.1 (M1)/0.01 (M2) | 0.5 | 0.25 |
| B | 0.1 (M1)/0.01 (M2) | - | 0.5 | 0.1 (M1)/0.01 (M2) |
| C | 0.5 | 0.5 | - | 1.0 |
| D | 0.25 | 0.1 (M1)/0.01 (M2) | 1.0 | - |

| **20-30 Myr** | A | B | C | D |
| --- | --- | --- | --- | --- |
| A | - | 0.1 (M1)/0.01 (M2) | 0.25 | 0.1 (M1)/0.01 (M2) |
| B | 0.1 (M1)/0.01 (M2) | - | 0.5 | 0.1 (M1)/0.01 (M2) |
| C | 0.25 | 0.5 | - | 1.0 |
| D | 0.1 (M1)/0.01 (M2) | 0.1 (M1)/0.01 (M2) | 1.0 | - |
