# Supplementary material for: Palaeoenvironmental Shifts Drove the Adaptive Radiation of a Noctuid Stemborer Tribe (Lepidoptera, Noctuidae, Apameini) in the Miocene
Source: PLoS One. 2012 Jul 31;7(7):e41377. doi: 10.1371/journal.pone.0041377 (PMC3409182; doi:10.1371/journal.pone.0041377)
Supplement: Table S3 — Selection of the best substitution models for each partition under jModelTest using AIC and BIC criterions. (DOCX) [file pone.0041377.s006.docx]

|  | **C01** | **CO1 Pos 1 & 2** | **C01 Pos 1** | **CO1 Pos 2** | **CO1 Pos 3** | **EF1a** | **EF1 Pos 1 & 2** | **EFIa Pos 1** | **EF1a Pos 2** | **EF1a Pos3** | **Pos1** | **Pos2** | **Pos3** |
| --- | --- | --- | --- | --- | --- | --- | --- | --- | --- | --- | --- | --- | --- |
| **AICc** | GTR+I+G | GTR+I+G | K80+G | JC | HKY+G | TIM1+I+G | TIM1+I+G | TrN+I+G | F81+I | TVM+G | HKY+I+G | TVM+G | GTR+I+G |
| **BIC** | GTR+I+G | GTR+I+G | TrN+I+G | F81+I | TVM+G | TIM1+I+G | TIM1ef+I+G | TrN+I+G | F81+I | TVM+G | F81+I+G | TVM+G | SYM+I+G |

**Table S3.** Selection of the best substitution model for each partition under jModelTest using corrected AIC (AICc) and BIC criterions.
