# Supplementary material for: Palaeoenvironmental Shifts Drove the Adaptive Radiation of a Noctuid Stemborer Tribe (Lepidoptera, Noctuidae, Apameini) in the Miocene
Source: PLoS One. 2012 Jul 31;7(7):e41377. doi: 10.1371/journal.pone.0041377 (PMC3409182; doi:10.1371/journal.pone.0041377)
Supplement: Table S4 — Reconstruction of ancestral host plant characters for Apameini using Lagrange. A, Amaryllidaceae; B, Cyperaceae; C, Iridaceae; D, Juncaceae; E, Liliaceae; F, Poaceae; G, Typhaceae; and H, Dicotyledons. The Poaceae are the ancestral host plant of Apameini. (DOCX) [file pone.0041377.s007.docx]

**Table S4.** Reconstruction of ancestral host plant characters for Apameini using Lagrange.

A, Amaryllidaceae; B, Cyperaceae; C, Iridaceae; D, Juncaceae; E, Liliaceae; F, Poaceae; G, Typhaceae; and H, Dicotyledons.

| Constrained Root | Global likelihood score |
| --- | --- |
| A | 134.4 |
| B | 129.4 |
| C | 131.3 |
| D | 134.2 |
| E | 134.4 |
| **F (Poaceae)** | **116.8** |
| G | 132.3 |
| H | 134.3 |

The Poaceae are the ancestral host plant of Apameini.
