# Supplementary material for: Palaeoenvironmental Shifts Drove the Adaptive Radiation of a Noctuid Stemborer Tribe (Lepidoptera, Noctuidae, Apameini) in the Miocene
Source: PLoS One. 2012 Jul 31;7(7):e41377. doi: 10.1371/journal.pone.0041377 (PMC3409182; doi:10.1371/journal.pone.0041377)
Supplement: Table S5 — Reconstruction of ancestral area for Apameini using Lagrange. A, Afrotropics; B, Nearctic; C, Palaearctic; and D, Oriental. The Palaearctic region is the ancestral area of Apameini for the three models. (DOCX) [file pone.0041377.s008.docx]

**Table S5.** Reconstruction of ancestral area for Apameini using Lagrange.

A, Afrotropics; B, Nearctic; C, Palearctic; and D, Oriental.

|  | Global likelihood score | | |
| --- | --- | --- | --- |
| Root | M0 | M1 | M2 |
| A | 99,04 | 106,8 | 112,7 |
| B | 101,6 | 107,8 | 113,6 |
| **C** | **96,31** | **102,9** | **108,6** |
| D | 106,2 | 115,3 | 121,2 |

The Palearctic region is the ancestral area of Apameini for the three models.
